# A new phenothiazine-based selective visual and fluorescent sensor for cyanide

**DOI:** 10.1186/s13065-019-0656-x

**Published:** 2020-01-07

**Authors:** Fatimah A. M. Al-Zahrani, Reda M. El-Shishtawy, Abdullah M. Asiri, Amerah M. Al-Soliemy, Khloud Abu Mellah, Nahed S. E. Ahmed, Abdesslem Jedidi

**Affiliations:** 1 Chemistry Department, Faculty of Science, King Khalid University, P.O.Box 9004, Abha, 61413 Saudi Arabia; 2 Chemistry Department, Faculty of Science, King Abdulaziz University, P.O. Box 80203, Jeddah, 21589 Saudi Arabia; 3 Dyeing, Printing and Textile Auxiliaries Department, Textile Research Division, National Research Centre, Dokki, Cairo 12622 Egypt; 4 Center of Excellence for Advanced Materials Research, King Abdulaziz University, Jeddah, 21589 Saudi Arabia; 5 Chemistry Department, Faculty of Science, Umm Al-Qura University, Mecca, Saudi Arabia

**Keywords:** Visual and fluorescent sensor, Phenothiazine, Cyanide, Nucleophilic addition, Detection limit, Intramolecular charge transfer

## Abstract

A new donor-π-acceptor derived from phenothiazine, namely 2-(2-((10-hexyl-10H-phenothiazin-3-yl)methylene)-3-oxo-2,3-dihydroinden-1-ylidene) malononitrile (PTZON) was synthesized and fully characterized, and its potential as a fluorescent sensor for cyanide anion was investigated. The PTZON showed a visible absorption band at 564 nm corresponds to an intramolecular charge transfer (ICT) and an emission band at 589 nm in CH_3_CN/H_2_O. The results of cyanide anion titration revealed ratiometric changes in both absorption and fluorescence spectra as a result of the nucleophilic addition of cyanide anion via Michael addition. The optical studies, FT-IR spectra, NMR, high-resolution mass, and DFT calculations confirmed the sensing mechanism. The selectivity of PTZON as a cyanide anion fluorescent sensor was proved in mixed solvent solutions, and the sensitivity was as low as 0.011 µM, which is far lower than the value allowed by the United States Environmental Protection Agency for drinking water (1.9 µM). Also, the detection limit of PTZON was assessed to be 3.39 μM by the spectrophotometric method. The binding stoichiometry between PTZON and cyanide anion was found to be 1:1 as evidenced by mass spectra. TLC silica-coated plates test strips demonstrated the fluorescent detection of cyanide anion.

## Introduction

Cyanide anion is notoriously toxic and deadly affect human beings because of its ability to bind to the active site of cytochrome oxidase and inhibit cellular respiration [1]. Cyanide anion is being involved in several industries such as metal plating, textile manufacture, and herbicides, and therefore, the awareness has to be taken not to load the environment by the effluents of these industries [2, 3]. At the point when cyanide enters the body by oral, inward breath, it applies its severe impacts by complexing with ferric iron molecules in metalloenzymes, bringing about histotoxic anoxia through restraint of cytochrome c oxidase [4, 5]. The WHO (world health organization) declared that the highest allowable level of cyanide anion concentration in the drinking water is 1.9 μM [6]. It has been reported that as little as 0.5–3.5 mg of cyanide per kilogram of human body weight can lead to death [7, 8]. Thus, monitoring and detection of cyanide anions are of great interest. Numerous techniques inclusive titrimetric [9], voltammetric [10], chromatographic strategies [11], electrochemical gadgets [12, 13], colorimetric [14–16] and fluorometric [17–25] have been used to estimate cyanide anions. Of the above-mentioned techniques, the fluorescence technique is considered the best due to its high sensitivity, fast response, low cost, and simple detection procedure [24–27]. In this interest, the development of fluorescent chemosensor for cyanide anions in aqueous solutions have been of growing interest [27–33]. The high nucleophilicity of cyanide anion inspired organic chemists to design and synthesize several chemosensors that function via nucleophilic addition mechanism [34–39]. Donor-π-acceptor (D-π-A) chromophores are known to have high tinctorial strength owing to the presence of an intramolecular charge transfer (ICT) band. The ICT band is easily tuned by varying the strength of the donor or acceptor or both. Such an interesting structural feature makes these compounds of great interest in various fields [40–45]. In this interest, the phenothiazine heterocyclic ring is a good electron donor in building chromophores of donor-π-acceptor type suitable as a fluorescent sensor, nonlinear optical material, and dye-sensitized solar cells [27, 46–48].

Here we present a new chemosensor derived from phenothiazine of donor-π-acceptor skeleton amenable for structural and optical changes upon cyanide addition with fast response. These changes are a consequence of breaking the ICT that exists between donor-π-acceptor. The selectivity and sensitivity of PTZON were investigated using UV–vis absorption and fluorescence. Additionally, the sensing mechanism was confirmed by DFT calculations, FT-IR, NMR and mass spectroscopies.

## Experimental

### General

All solvents and reagents were of the highest purity available, purchased from Sigma-Aldrich Company and used as received. ^1^H and ^13^ C NMR spectra were recorded in CDCl_3_ and DMSO-d_6_ solution on a Bruker Avance 600 and 400 MHz spectrometer. Infrared spectra were performed on PerkinElmer spectra 100 FTIR spectrometer. Mass spectroscopy was performed using Agilent GC 7000 mass spectrometers. UV absorption spectra were determined in different solvents on Shimadzu UV–VIS Spectrophotometer. Fluorescence spectra were recorded on PerkinElmer LS 55 Fluorescence Spectrometer.

### Synthesis and characterization

#### 2-(2-((10-hexyl-10H-phenothiazin-3-yl)methylene)-3-oxo-2,3-dihydroinden-1-ylidene)malononitrile (PTZON)

A mixture of **2** [49] (3 mmol) and 3-dicyanovinylindan-1-one (6 mmol) in basic ethanol solution (7 ml) was stirred at room temperature overnight, filtered off and crystallization from cyclohexane to afford 80% yield. M. p. 89–90 °C; ^1^H NMR (600 MHz, DMSO-d_6_) δ 0.84 (t, 3H,CH_3_), 1.25 (m, 4H, CH_2_), 1.27 (m, 2H, CH_2_), 1.70 (m, 2H, CH_2_), 3.96 (t, 2H, CH_2_-N), 7.02 (t, 2H, Ar–H), 7.09 (d, 2H, Ar–H), 7.17 (m, 4H, Ar–H), 7.24 (m, 2H, Ar–H), 7.60 (s, H, vinylic proton),7.73 (d,1H, Ar–H).^13^C NMR (125 MHz, DMSO-d_6_) δ 14.19, 22.81, 26.73, 26.98, 27.14, 31.59, 48.52, 70.52, 114.56, 114.84, 116.15, 124.22, 124.25, 125.34, 126.66, 127.77, 133.55, 134.80, 135.37, 136.67, 137.62, 139.91, 146.54, 150.69, 162.66, ESI–MS m/z [M]^+^calc 487.61 found 486., IR $\nu$/cm^−1^: C–H aliphatic 2925, 2851, CN 2214, C=O 1739, C=C 1694.

### General spectroscopic procedures

#### Method

A solution of PTZON (2 × 10^−5^ M) in acetonitrile–water (90:10) was titrated with increments of aqueous KCN (2 × 10^−3^ M) and were monitored by UV–visible and fluorescence methods. Titration experiments were carried out in 10-mm quartz cell at room temperature. (λ_ex_ = 500 nm, λ_em_ = 588 nm).

#### Selectivity

The selectivity experiment was done by monitoring the fluorescence intensity changes of PTZON (2 × 10^−5^ M) in acetonitrile–water (90:10) at 588 nm (λ_ex_ = 500 nm) upon addition of various anions at the concentrations indicated below the figure.

#### Detection limit

The following equation calculated the limit of detection (**LOD**); **LOD = 3S/ρ**, where **S** is the standard deviation of blank measurements (10 runs), **ρ**, is the slope between intensity versus sample concentration.

### Computational details

Geometries of PTZON and PTZON-CN^−^ were optimized in the vacuum through Density Functional Theory (DFT) via the spin-restricted Kohn–Sham formalism and the hybrid B3LYP functional [50, 51] using the valence double zeta basis set 6-31G(d) [52]. In all cases, frequency calculations were performed in order to confirm the nature of the stationary points (minima with no imaginary frequency). The UV–Vis parameters (maximum wavelength, electronic excitation energies, and oscillator strengths) of the studied compounds have been calculated with the time-dependent density functional theory (TD-B3LYP) at the 6-31G(d) level of theory [53, 54] in order to evaluate the sensing effect made by CN^−^ ion. All the detailed calculations were carried out through the facilities provided by the Gaussian09 package [55].

## Results and discussion

### Synthesis of PTZON

**Scheme 1:**
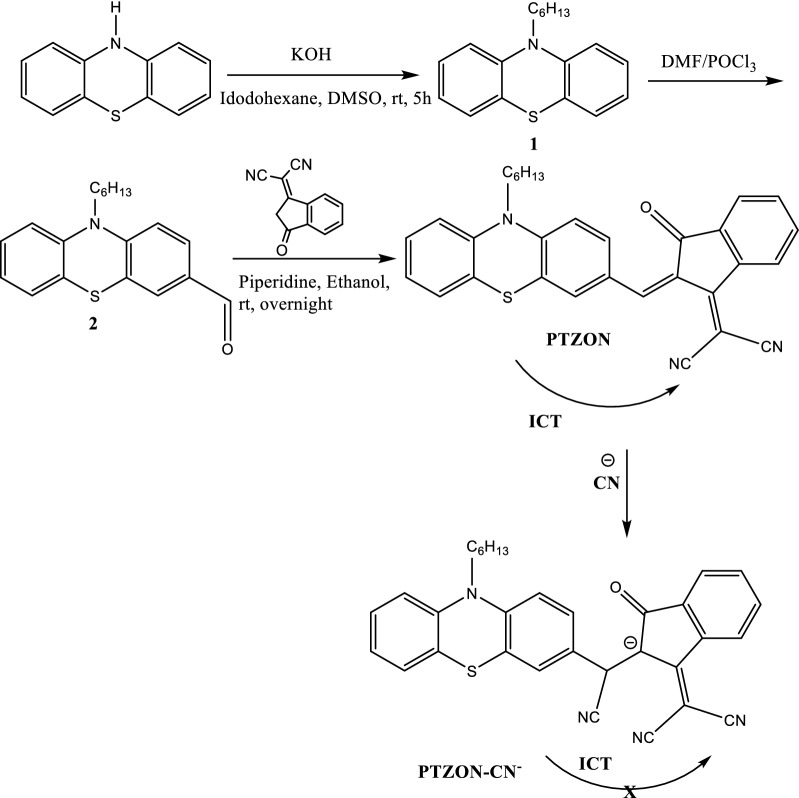
Synthesis of PTZON and the sensing mechanism

As shown in Scheme 1, PTZON was synthesized by the Knoevenagel condensation of 10-hexyl-10H-phenothiazine-3-carbaldehyde and 3-dicyanovinylindan-1-one. The molecular structure of the PTZON was confirmed by FTIR, ^1^H NMR, ^13^C NMR, and mass spectra.

### UV–vis absorption and fluorescence properties

**Fig. 1:**
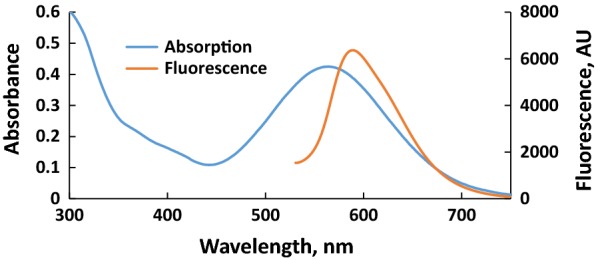
Absorption and fluorescence spectra of **PTZON** (2 × 10^−5^ M) in acetonitrile–water (90:10)

UV–vis absorption and fluorescence spectra of PTZON in acetonitrile–water (90:10) are presented in Fig. 1 to reveal the maximum wavelength of absorption and fluorescence at 564 and 589 nm, respectively. The absorption band at 564 nm is due the ICT presents in the molecule with a molar extinction coefficient about 2.1 × 10^4^ M^−1^cm^−1^.

**Fig. 2:**
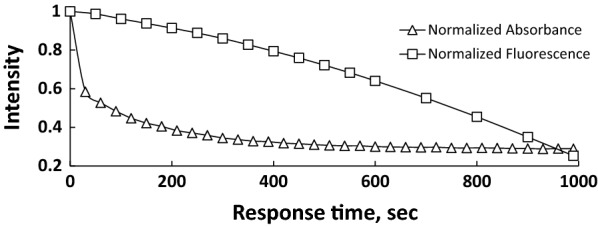
Response time for the detection of cyanide anion in acetonitrile–water (90:10)

**Fig. 3:**
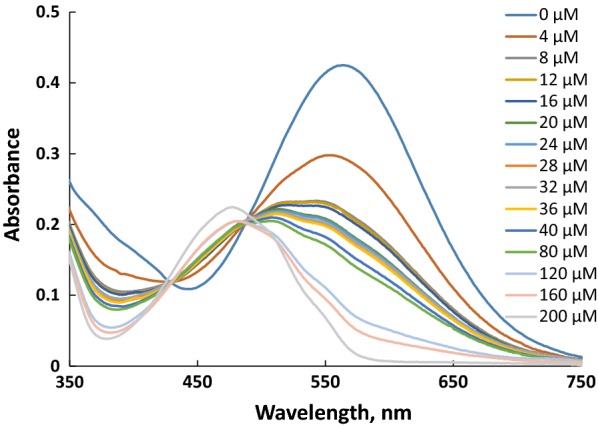
Absorption spectra of **PTZON** (2 × 10^−5^ M) upon the addition of cyanide anion at different concentration in acetonitrile–water (90:10)

**Fig. 4:**
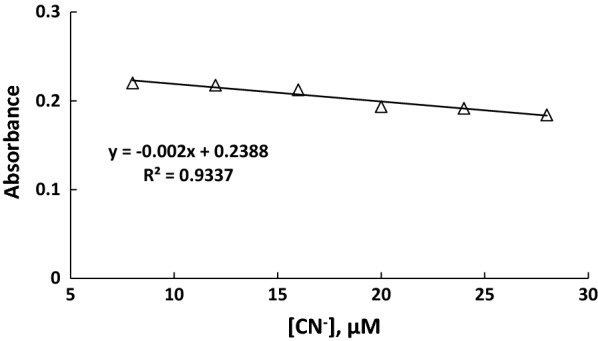
Absorption intensity calibration curve of **PTZON** (2 × 10^−5^ M) as a function of cyanide anion concentration in acetonitrile–water (90:10)

**Fig. 5:**
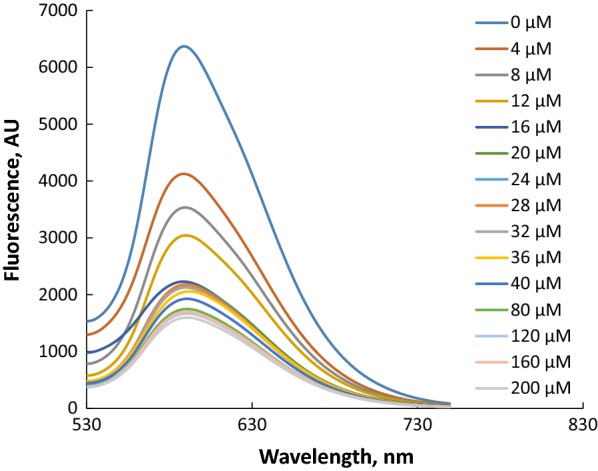
Fluorescence spectra of **PTZON** (2 × 10^−5^ M) upon the addition of cyanide anion at different concentration in acetonitrile–water (90:10). The fluorescence intensity was measured at 25 °C (λ_ex_ = 500 nm, λ_em_ = 588 nm)

**Fig. 6:**
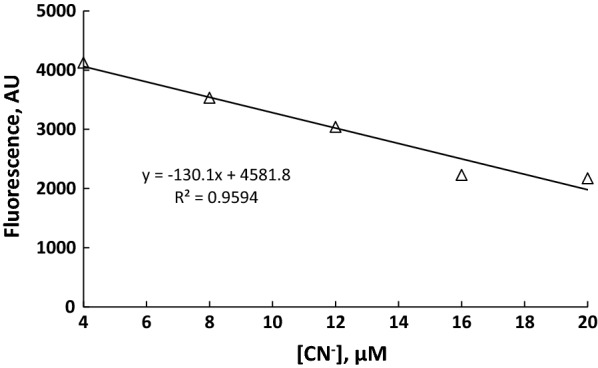
Fluorescence intensity calibration curve of **PTZON** (2 × 10^−5^ M) as a function of cyanide anion concentration in acetonitrile–water (90:10)

Response time shown in Fig. 2 for both UV–visible and fluorescence indicates that the response is fast, and about 3 min was sufficient time to reach the plateau of change, and therefore, 3 min was considered as the response time through the present study. The result of cyanide anion titration, as shown in Figs. 3, 4, 5, 6, concluded the value of detection limit (LOD) to be 3.39 and 0.011 μM by spectrophotometric and spectrofluorophotometric methods, respectively.

### Selectivity studies

**Fig. 7:**
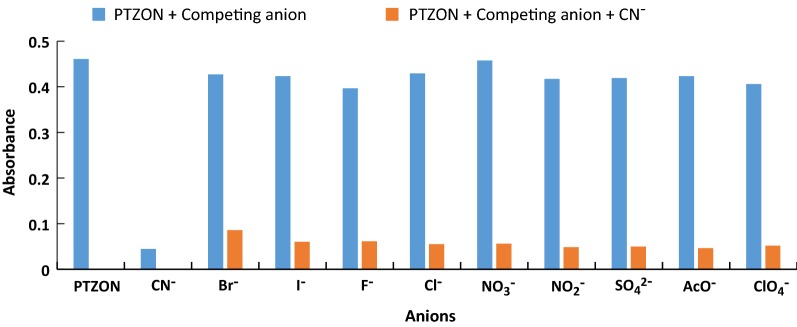
The absorbance changes of **PTZON** (2 × 10^−5^ M) in acetonitrile–water (90:10) in the presence of competing anions

**Fig. 8:**
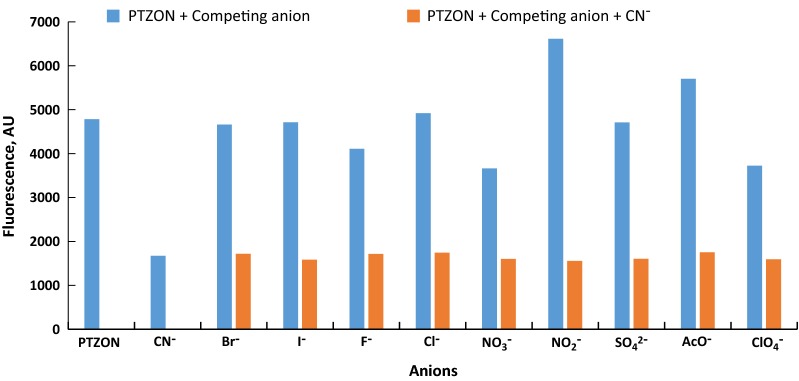
The fluorescence intensity changes of **PTZON** (2 × 10^−5^ M) in acetonitrile–water (90:10) in the presence of competing anions

**Fig. 9:**
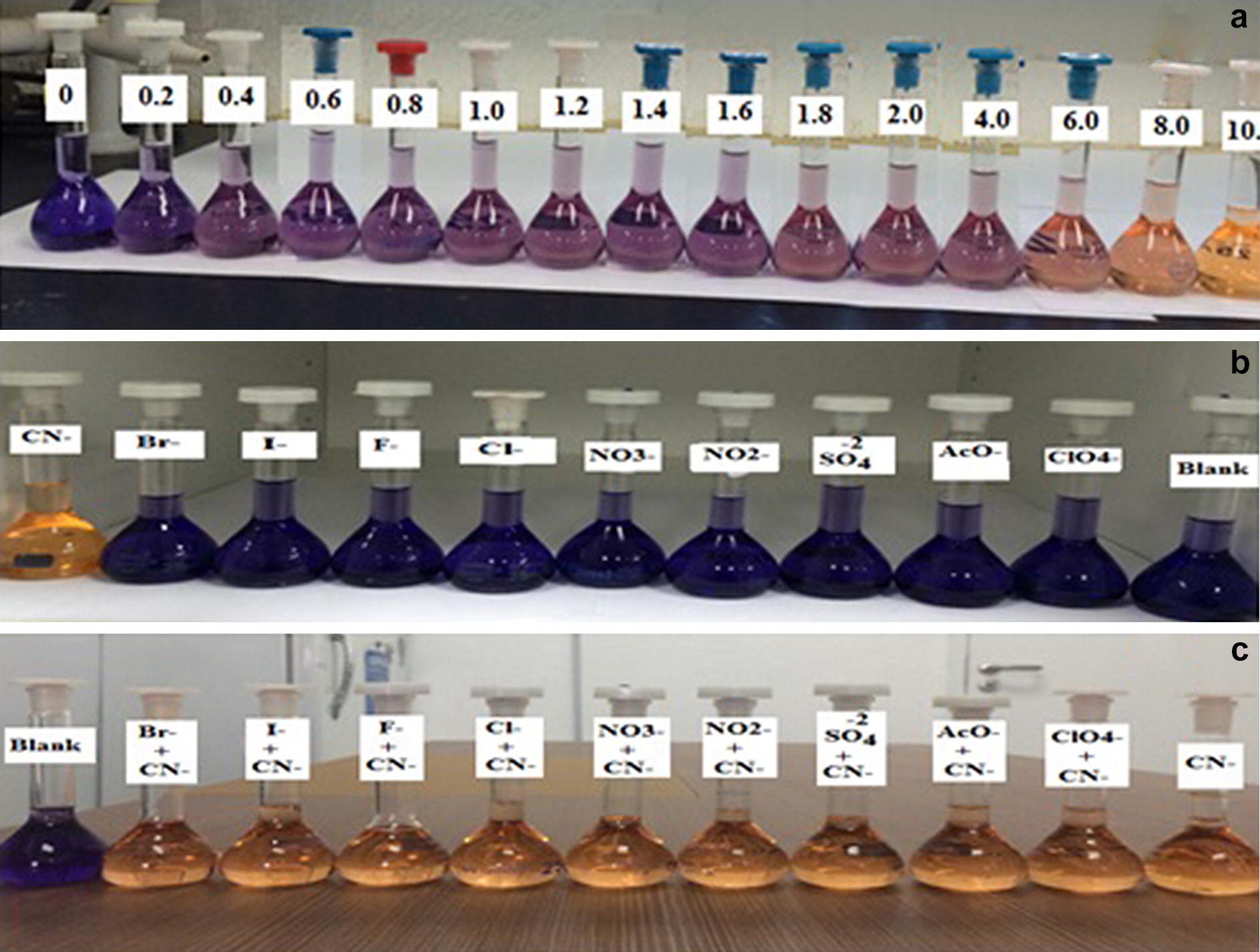
The color changes upon gradual addition of different equiv. of cyanide anion (**A**) upon addition of 10 equiv. of different anions (**B**) and upon mixing 10 equiv. of cyanide anion and another competing anion (5 equiv. + 5 equiv. each) (**C**). **A** Image of CN^−^ responsive PTZON (from left to right: 0–10 equiv.); **B** in the presence of 10 equiv of different anions (from left, CN^−^, Br^−^, I^−^, F^−^, Cl^−^, NO_3_^−^, NO_2_^−^, SO_4_^2−^, AcO^−^, ClO_4_^−^, blank); **c** in the presence of 5 equiv. of CN^−^ and 5 equiv. of different anions (from left, blank, Br^−^, I^−^, F^−^, Cl^−^, NO_3_^−^, NO_2_^−^, SO_4_^2−^, AcO^−^, ClO_4_^−^, CN^−^)

The selectivity of a chemosensor is an essential property for its possible application. Therefore, it was desired to investigate the selectivity of PTZON for cyanide anion detection in the presence of other anions. For this purpose, the fluorescence of PTZON solution alone, after being mixed with different anions of ten equiv., and after being mixed with different anions in the presence of cyanide anion in 5 + 5 equiv. in CH_3_CN/H_2_O solutions was followed after 3 min of response time. Interestingly, PTZON revealed high selectivity toward CN^−^ than other anions, as shown in the figure. Although the other anions were used in large excess (10 equiv.) compared with cyanide anion (5 equiv.) yet the fluorescence of PTZON was slightly changed compared with the huge change made by cyanide anion. Also, the test of interference anions confirmed that the huge change observed was due to the selectively of PTZON toward cyanide anion. Figures 7, 8 and 9 show the selectivity of PTZON.

### Sensing mechanism

**Fig. 10:**
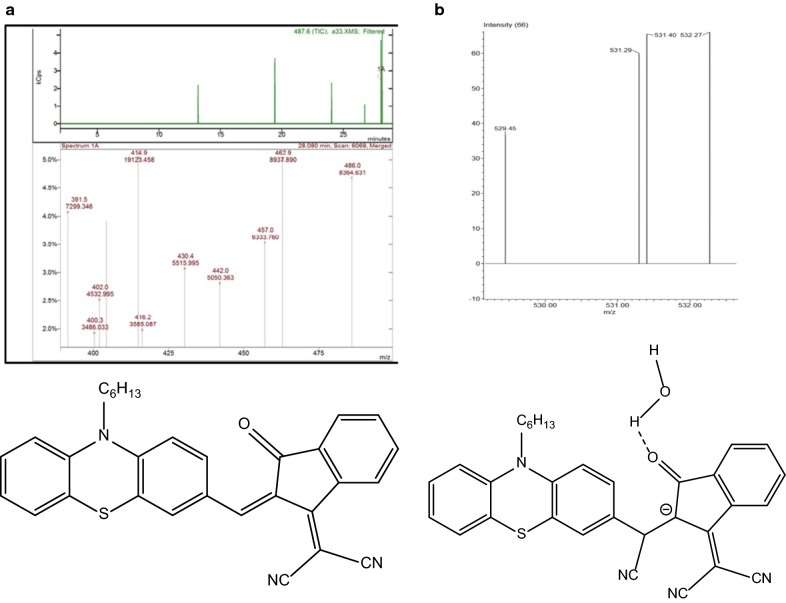
LC–MS of **PTZON** before (**a**) and after addition of CN^−^ (**b**)

The sensing mechanism was thought to be via Michael addition reaction of cyanide anion on β-vinylic carbon and to prove this mechanism; the high-resolution mass spectra of PTZON before and after cyanide anion addition was made. As shown in Fig. 10 the mass of PTZON (A) m/z: calcd for C_31_H_25_N_3_OS: 487.17 [M]^+^ that found: 486.0 [M–H]^+^ has become after cyanide addition (B): 531.19 [M+CN+H_2_O]^+^, indicating that the addition is 1:1 stoichiometry of PTZON and cyanide anion.

**Fig. 11:**
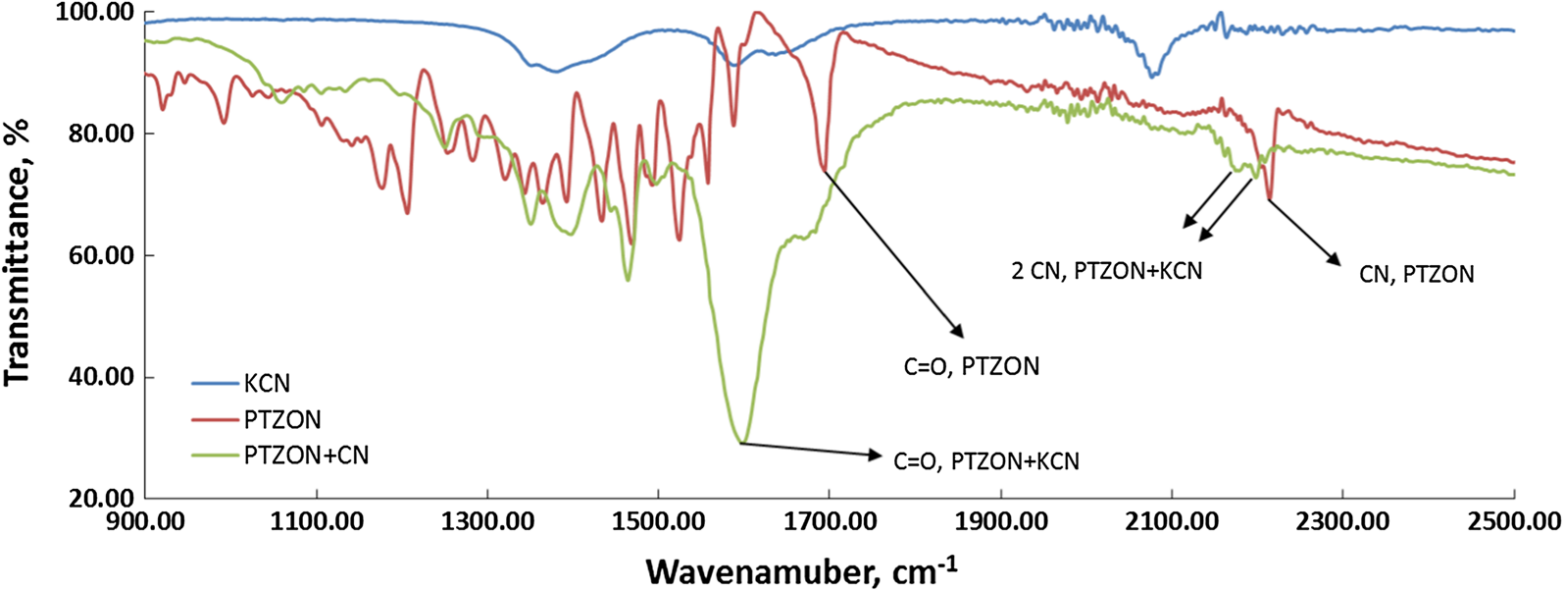
FTIR of **PTZON** in absence and presence of cyanide anion

On the other hand, the FTIR tool was also used to confirm the structure after cyanide anion addition on PTZON. The most striking difference, as shown in Fig. 11 is the disappearance of a sharp peak at 1706 cm^−1^ due to C=O present in PTZON before cyanide anion addition and the appearance of a broad peak at 1600 cm^−1^ due to hydrogen-bonded C=O after cyanide anion addition. Additionally, the sharp peak that appears at 2200 cm^−1^ due to CN present in PTZON has become shifted to a lower frequency to appear at 2214 cm^−1^ with the appearance of another CN peak at 2179 cm^−1^ after cyanide anion addition.

### DFT studies

Computational study of the PTZON before and after cyanide anion addition would further confirm the sensing mechanism. For this purpose, geometry optimization of both forms, followed by energy computation, was made using the computational details mentioned in the experimental section.

#### Geometrical analysis

**Fig. 12:**
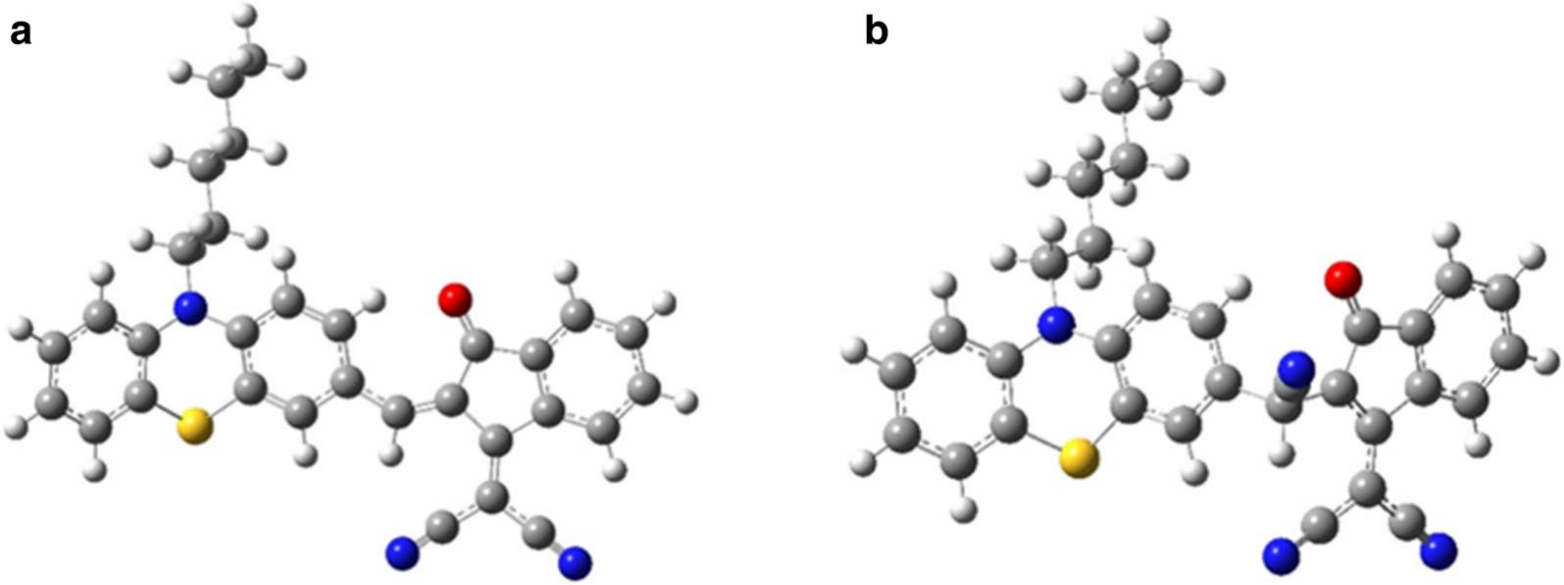
Optimized structures PTZON (**a**) and PTZON-CN^−^ (**b**) with B3LYP/6-31G(d) method

PTZON structure is originally a flat structure with phenothiazine and indanone moieties. The substitution of the two carbon atoms in the para position of the middle ring in the phenothiazine ring by N and S atoms has generated a butterfly-like structure (Fig. 12a). We have checked the stability of this structure by frequency calculations, and no negative frequency means the structure is a low energy structure. We have added cyanide at the level of the trigonal carbon atom linking the phenothiazine and the indanone moieties (sp2 hybridization). After addition, the structure becomes tetragonal at the same point, and the carbon goes for sp^3^ hybridization with a negative charge at the neighboring atom (see Fig. 12b).

#### Vibrational analysis

**Fig. 13:**
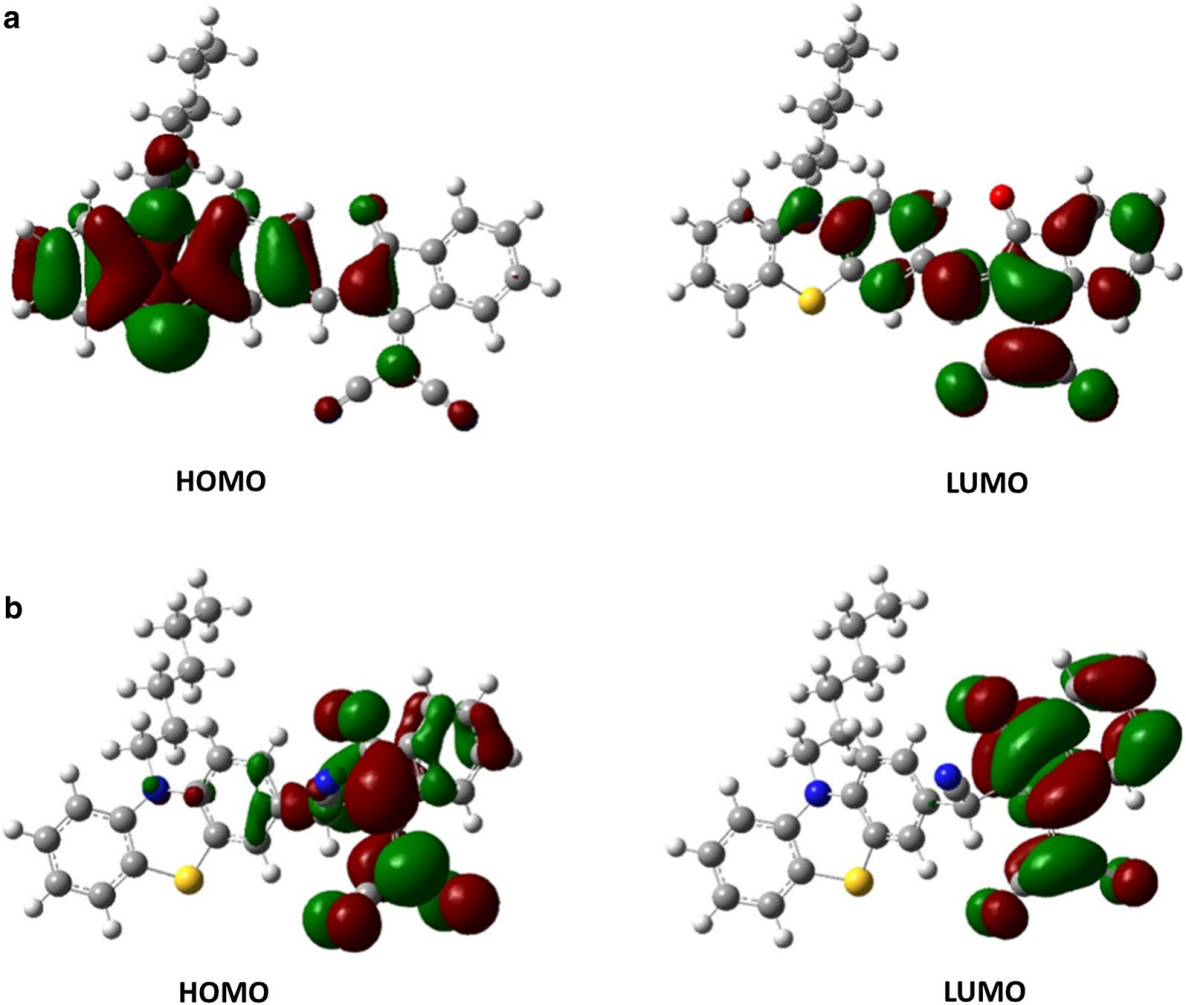
Contour plots of frontier molecular orbitals (isovalue = 0.02) of compound PTZON (**a**) at the ground state geometry and PTZON-CN^−^ (**b**) at the first excited singlet state geometry

The two structures showed that C=O vibrate at 1774 cm^−1^, for PTZON, which is reduced in the presence of CN^−^ to reach 1723 cm^−1^ (PTZON-CN^−^). The same behavior happened for the cyanide groups actually present in the initial structure (PTZON) with a frequency 2314–2333 cm^−1^ that goes to 2268–2298 cm^−1^ with one additional peak at 2350 cm^−1^ for the added CN^−^ in PTZON-CN^−^ (Table 1). The frontier molecular orbitals (FMOs) are represented mainly by the Highest Occupied Molecular Orbitals (HOMOs) and the Lowest Unoccupied Molecular Orbitals (LUMOs). Figure 13 shows the FMOs of the unbound and CN-bound PTZON simulated using the B3LYP/6-31G(d) level of theory.

**Table 1:** Vibrational frequency analysis using B3LYP/6-31G(d) method

Structure	PTZON	PTZON-CN^−^
Assignment	Frequency (cm^−1^)	
C=O	1774	1723
C≡N	(2314–2333)	(2268–2298)–2350^*^

The frequency between parenthesis are those for C–N bond present in both PTZON and PTZON-CN^−^

The frequency with a asterisk correspond to the added CN^−^ to the PTZON that is why it does not appear in the former one (PTZON)

#### Charge transfer and UV–visible analysis

**Fig. 14:**
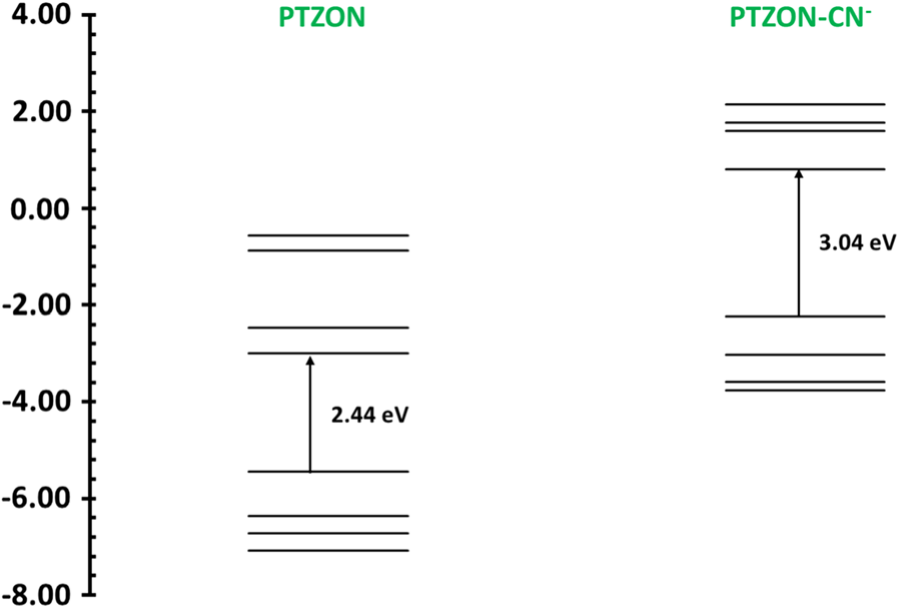
Molecular orbital energy level diagram of PTZON (left) and PTZON-CN^−^ (right)

The electron density in HOMO is mainly distributed on the phenothiazine ring, and the electron density in LUMO is distributed on the indanone moiety. This indicates the possible charge transfer from phenothiazine (donor) to the indanone moiety (acceptor). PTZON shows a strong absorption band at 581 nm, which corresponds to the charge transfer band. As expected, all the HUMO and LUMO energy levels were raised after the formation of PTZON-CN^−^ adduct, and the energy bandgap has increased from 2.44 to 3.04 eV (Fig. 14). This increment in the energy gap value implies the breakage of π-conjugation between phenothiazine and indanone moieties. Thus the ICT process was stopped. This is consistent with the experimental observation with the complete disappearance of ICT band at 588 nm when PTZON-CN^−^ adduct is formed. UV–vis spectra originate from π → π* and n → π* electronic transitions in π-conjugated organic compounds [56]. Table 2 lists the maximum absorption wavelengths of PTZON and PTZON-CN^−^ adduct using the B3LYP functionals and 6-31G(d) basis set. Excellent agreement was obtained between the experimental values of the unbound and bound PTZON and our theoretical simulation values (S19).

**Table 2:** Computed absorbance (E_abs_) and emission wavelength (λ_abs_), oscillator strengths (f) and molecular orbital (MO) compositions for the low-lying excited singlet states of compound PTZON and PTZON-CN^−^

State	E_abs_/em	λ_abs_/em	f	MO
PTZON-S1	2.1343	580.91	0.2915	H-0 --> L+0 (98%)
PTZON-S2	2.5695	482.52	0.0671	H-0 --> L+1 (96%)
PTZON-S3	2.9716	417.22	0.3952	H-1 --> L+0 (93%)
PTZON-CN	2.6879	461.26	0.1057	H-0 --> L+0 (99%)

### Test strip

**Fig. 15:**
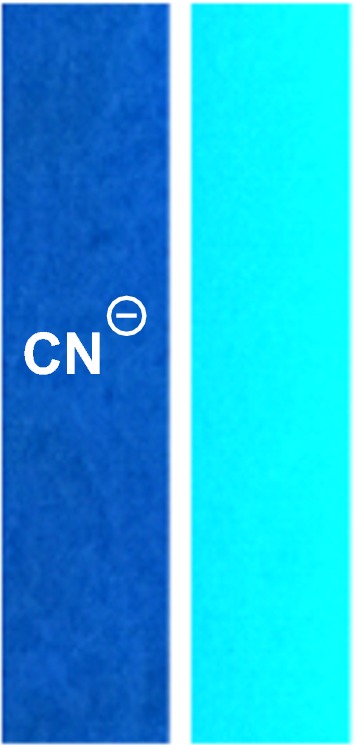
Color changes of the test strips under UV lamp containing **PTZON** treated with cyanide anion (left) and untreated (right)

As a demonstration for possible practical application, a test strip from TLC was dipped in PTZON solution (10^−3^ M solution in acetonitrile) and air-dried. This process of dipping and air-drying was repeated three times to colorize the strip. Half of the test strip was immersed in aqueous cyanide solution, and the image was taken under UV-lamp for the immersed and non-immersed strip to show the color difference clearly. The demonstrated data prove the suitability of utilizing a simple test strip of PTZON for the fast detection of cyanide anion (Fig. 15).

## Conclusion

A new PTZON chemosensor based-Michael addition mechanism of sensing cyanide anion was synthesized and characterized. PTZON was a turn-off fluorescent sensor of cyanide anion, and the estimated LOD was 0.011 µM, which is far lower than the level allowed by WHO (1.9 µM). The optical studies, FT-IR spectra, NMR, high-resolution mass, and DFT calculations confirmed the sensing mechanism. A simple test strip demonstrated the suitability of using PTZON with a fast response. Hence, this study introduces a new and simple way for the development of a highly sensitive cyanide sensor viable for application qualitatively by naked-eye detection and quantitatively by fluorescence technique. Further studies are in progress for devising new chemosensors suitable for sensing applications.

## Data Availability

The datasets used and/or analysed during the current study are available from the corresponding author on reasonable request.

## Abbreviations

^1^ H NMR: proton nuclear magnetic resonance
^13^C NMR: carbon-13 nuclear magnetic resonance
FT-IR: Fourier transform infrared spectroscopy
DFT: density functional theory
FMOs: frontier molecular orbitals
HOMOs: highest occupied molecular orbitals
LUMOs: lowest unoccupied molecular orbitals
ICT: intramolecular charge transfer
UV–VIS: ultraviolet-visible
PTZON: 2-(2-((10-hexyl-10H-phenothiazin-3-yl)methylene)-3-oxo-2,3-dihydroinden-1-ylidene) malononitrile
PTZON-CN^−^: 2-(2-((10-hexyl-10H-phenothiazin-3-yl)methylene)-3-oxo-2,3-dihydroinden-1-ylidene) malononitrile after addition of cyanide anion
DMSO-d_6_: deuterated dimethyl sulfoxide
CH_3_CN: acetonitrile
TLC: thin layer chromatography
WHO: world health organization
LOD: limit of detection
